# 2509. Association of Non-Alcoholic Fatty Liver Disease with HBV, HAV, and HEV Exposure

**DOI:** 10.1093/ofid/ofad500.2127

**Published:** 2023-11-27

**Authors:** Stephanos Vassilopoulos, Athanasios Vassilopoulos, Gregorio Benitez, Matthew Kaczynski, Fadi Shehadeh, Markos Kalligeros, Eleftherios Mylonakis

**Affiliations:** Warren Alpert Medical School of Brown University, Rhode Island Hospital, Providence, RI, Providence, Rhode Island; Warren Alpert Medical School of Brown University, Rhode Island Hospital, Providence, RI, Providence, Rhode Island; Warren Alpert Medical School of Brown University, Rhode Island Hospital, Providence, RI, Providence, Rhode Island; Warren Alpert Medical School of Brown University, Rhode Island Hospital, Providence, RI, Providence, Rhode Island; Houston Methodist Research Institute, Houston, TX, Houston, Texas; Warren Alpert Medical School of Brown University, Rhode Island Hospital, Providence, RI, Providence, Rhode Island; Houston Methodist Hospital, Houston, TX, Houston, Texas

## Abstract

**Background:**

Non-alcoholic fatty liver disease (NAFLD) is a leading cause of chronic liver disease. The association between prior hepatitis B virus (HBV), hepatitis A virus (HAV), and hepatitis E virus (HEV) infection and NAFLD has not been elucidated. The National Health and Nutrition Examination Survey (NHANES) aims to assess the health and nutritional status of the population across the United States.

**Methods:**

By using the 2017-2020 NHANES, we evaluated the age-adjusted prevalence of prior HBV, HAV and HEV infection among participants with NAFLD, as well as the age adjusted prevalence of NAFLD, high risk non-alcoholic steatohepatitis (NASH) and liver fibrosis among participants with prior HBV infection. We then performed multivariable logistic regression analyses using adjusted odds ratios (aOR) to examine the association of prior HBV, HAV and HEV infection with NAFLD, high risk NASH, and liver fibrosis.

**Results:**

We identified 2565 individuals with anti-HBc serology results, 1480 unvaccinated participants with anti-HAV results, and 2561 participants with anti-HEV results. Among participants with NAFLD, the age-adjusted prevalence of prior HBV, HAV and HEV infection was 3.48%, 32.08% and 7.45%, respectively. The age-adjusted prevalence of NAFLD, high-risk NASH and fibrosis among participants with prior HBV infection was 33.59%, 3.36%, and 8.00%, respectively. Prior infection with HBV, HAV and HEV was not associated with NAFLD or high-risk NASH. Participants with anti-HBc and anti-HAV seropositivity were more likely to have significant fibrosis compared to participants with negative anti-HBc and anti-HAV serology [aOR: 1.53 (95% CI, 1.05 – 2.23) and 1.69 (95% CI, 1.16 – 2.47), respectively].

**Figure:**
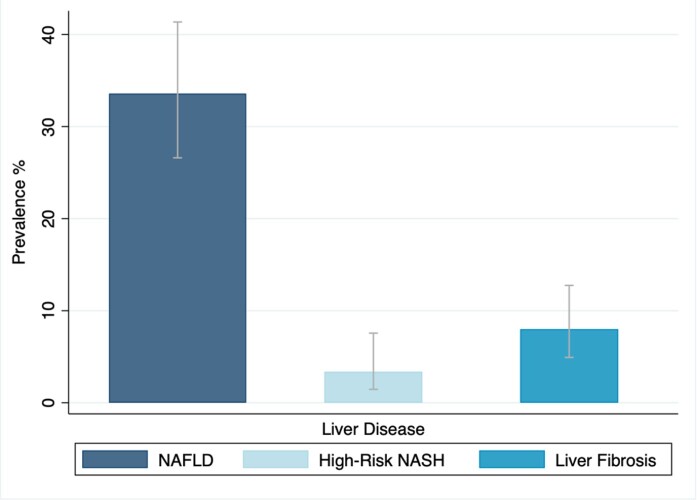

Age-adjusted prevalence of NAFLD, high-risk NASH and liver fibrosis among participants with positive anti-HBc serology

**Figure:**
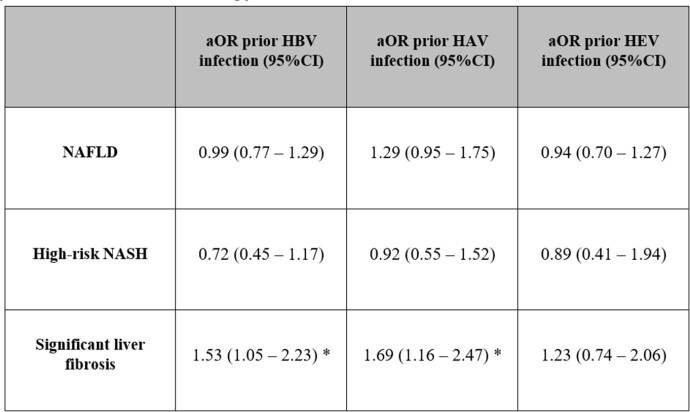

Adjusted odds ratio of prior viral hepatitis exposure

**Conclusion:**

Participants with a history of HBV or HAV infection exhibit greater odds of significant liver fibrosis compared to those without HBV or HAV exposure. To limit disease-related outcomes, healthcare providers should prioritize vaccination efforts and use an individualized strategy for NAFLD in patients with prior viral hepatitis, including HBV or HAV infection.

**Disclosures:**

**Eleftherios Mylonakis, MD, PhD**, BARDA: Grant/Research Support|Basilea: Advisor/Consultant|Chemic Labs/KODA Therapeutics: Grant/Research Support|Cidara: Grant/Research Support|Leidos Biomedical Research Inc./NCI: Grant/Research Support|NIH/NIAID: Grant/Research Support|NIH/NIGMS: Grant/Research Support|Pfizer: Grant/Research Support|Regeneron Pharmaceuticals, Inc.: Grant/Research Support|SciClone Pharmaceuticals: Grant/Research Support

